# Correction: Real-time structural dynamics of the ultrafast solvation process around photo-excited aqueous halides

**DOI:** 10.1039/d4sc90163k

**Published:** 2024-08-16

**Authors:** Verena Markmann, Jaysree Pan, Bianca L. Hansen, Morten L. Haubro, Amke Nimmrich, Philipp Lenzen, Matteo Levantino, Tetsuo Katayama, Shin-ichi Adachi, Simone Gorski-Bilke, Friedrich Temps, Asmus O. Dohn, Klaus B. Møller, Martin M. Nielsen, Kristoffer Haldrup

**Affiliations:** a Technical University of Denmark Anker Engelunds Vej 1 2800 Lyngby Denmark vmark@dtu.dk; b Department of Chemistry and Molecular Biology, University of Gothenburg Gothenburg Sweden; c European Synchrotron Radiation Facility CS40220 Grenoble 38043 Cedex 9 France; d Japan Synchrotron Radiation Research Institute Kouto 1-1-1 Sayo Hyogo 679-5198 Japan; e RIKEN SPring-8 Center 1-1-1 Kouto Sayo Hyogo 679-5148 Japan; f Institute of Materials Structure Science, High Energy Accelerator Research Organization (KEK) 1-1 Oho Tsukuba Ibaraki 305-0801 Japan; g Department of Materials Structure Science, School of High Energy Accelerator Science 1-1 Oho Tsukuba Ibaraki 305-0801 Japan; h Christian-Albrechts-University Kiel Olshausenstr. 40 24098 Kiel Germany; i Science Institute, University of Iceland 107 Reykjavík Iceland

## Abstract

Correction for ‘Real-time structural dynamics of the ultrafast solvation process around photo-excited aqueous halides’ by Verena Markmann *et al.*, *Chem. Sci.*, 2024, **15**, 11391–11401, https://doi.org/10.1039/D4SC01912A.

The authors regret that the equation in the second paragraph of Section 3 (Time-resolved X-ray solution scattering) on page 11393 was incorrect in the original article. The correct equation is shown below:
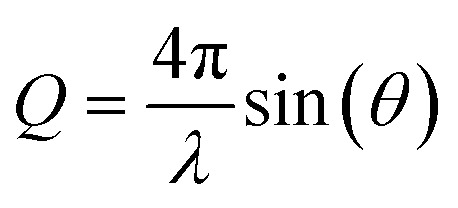


The Royal Society of Chemistry apologises for these errors and any consequent inconvenience to authors and readers.

